# Fine Particulate Exposure During Pregnancy Impacts on Perinatal Complications in Deeply Phenotyped Preterm Infants With Significant Immaturity

**DOI:** 10.1002/pul2.70320

**Published:** 2026-05-13

**Authors:** Caroline Johansson, Yvonne Kraus, Juan David Henao Sanchez, Benjamin Schubert, Kathrin Wolf, Carola Voss, Tobias Stoeger, Tanja Seegmüller, Andreas W. Flemmer, Kai Förster, Marie Standl, Anne Hilgendorff

**Affiliations:** ^1^ Dr. von Hauner Children's Hospital, Hospital of the Ludwig Maximilian Universität München Munich Germany; ^2^ Computational Health Center, Helmholtz Munich Member of the German Center for Lung Research (DZL) Munich Germany; ^3^ Department of Mathematics Technische Universität München, Garching bei München Munich Germany; ^4^ Institute of Epidemiology, Helmholtz Zentrum München GmbH German Research Center for Environmental Health Neuherberg Germany; ^5^ Institute of Lung Health and Immunity (LHI), Helmholtz Zentrum München GmbH, Comprehensive Pneumology Center (CPC‐M), Germany Member of the German Center for Lung Research (DZL); ^6^ Hannover Medical School, Clinic for Cardiac, Thoracic, Transplantation and Vascular Surgery, Leibniz Research Laboratories for Biotechnology and Artificial Organs (LEBAO), Biomedical Research in Endstage and Obstructive Lung Disease Hannover (BREATH) Member of the German Center for Lung Research (DZL); ^7^ Division of Neonatology, Dr. von Hauner Children's Hospital LMU University Hospital Munich Germany; ^8^ Member of the German Center for Lung Research (DZL) and German Center for Child and Adolescent Health (DZKJ) Munich Germany; ^9^ School of Medicine and Health Sciences, Department of Paediatrics, Section of Neonatology, Pediatric Intensive Care, Pediatric Cardiology, Pediatric Pneumology and Allergology and Research Centre Neurosensory Science Carl von Ossietzky University Oldenburg Oldenburg Germany

**Keywords:** air pollution, birth weight, fine particulate matter, preeclampsia, preterm infant

## Abstract

The identification of ubiquitous risk factors determining long‐term morbidity is crucial in infants born prematurely when aiming to develop prevention strategies. In a cohort of deeply phenotyped infants born before 32 weeks gestational age, we successfully demonstrate that exposure to fine particulate matter during pregnancy is associated with increased odds of preeclampsia and altered birth weight percentiles, highlighting potential underlying effects on vascular and metabolic pathology in relation to the degree of immaturity.

## Trial Registration

German Registry for Clinical Studies (DRKS00004600).

## 1 Introduction

Long‐term morbidity after premature birth is of significant relevance for patients and caretakers. Understanding the impact of environmental insults on morbidity development is crucial for the design of preventive strategies. Next to the postnatal impact of oxygen toxicity, mechanical ventilation, and infections on organ development in the preterm infant, the prenatal exposure to airborne toxicants has gained increasing interest, especially when considering multiple‐hit events as the driver of morbidity. Studies on the effects of environmental pollutants have, however, mainly focused on overall pregnancy outcome [1], next to studies unraveling effects on cardiopulmonary health [2] and metabolic diseases [3] in the general population. Deeper insight into the impact of environmental toxicants on detailed neonatal outcome is missing, as these questions require deeply phenotyped clinical cohorts that can build on diagnostic details beyond the level of epidemiological studies [4].

We therefore investigated the effect of prenatal residential exposure levels to airborne toxicants, that is, black carbon (BC), fine particulate matter (PM2.5), nitrogen dioxide (NO_2_), and ozone (O_3_), on perinatal outcome in a cohort of infants with significant immaturity comprehensively monitored from birth to discharge (Attention to Infants at Respiratory Risks [AIRR] cohort [5]).

## 2 Methods

### 2.1 Study Population and Clinical Monitoring

Between 2012 and 2020, sixty preterm infants born < 32 weeks gestational age (GA) at the Perinatal Center of the Ludwig‐Maximilian University (GA: 27.1 ± 2.3 weeks; birth weight: 440–1770 g, 908.3 ± 317.3 g; mean and standard deviation, 60% male) were prospectively included in the study upon informed parental consent (AIRR [5], LMU Ethic Board #195‐07, German Registry for Clinical Studies DRKS00004600). The perinatal course was monitored in all study infants until discharge: 13 cases (22%) were diagnosed with preeclampsia during pregnancy, 3 cases (5%) were diagnosed as small for GA (birth weight < 10th percentile). Postnatally, the following case numbers were diagnosed with the respective complications: early‐onset infection (EOI [6]): 12 cases (20%); intraventricular hemorrhage [IVH] ≥ grade 3: 3 [5%]; retinopathy of prematurity [ROP] ≥ grade 3: 7 cases [12%]; respiratory distress syndrome [RDS] [7] ≥ grade 3: 26 cases [43%]; bronchopulmonary dysplasia [BPD] [8]): 45 cases (75% total, 40% moderate/severe). Antenatal glucocorticosteroid treatment was administered in 51 cases (85%), postnatal steroids in 28 cases (47%). Median oxygen supplementation was 61 (0–504), IPPV or mechanical ventilation 3 (0–43), NIPPV or CPAP 42 (0–102) days (median and range each). Overall NICU stay was 66 (21–152) days. Clinical parameters see File S1.

### 2.2 Air Pollutant Exposure

Residential exposure levels to four ambient air pollutants, that is, BC, PM2.5, NO_2_, and O_3_ during pregnancy were derived from and use regression models developed for Western Europe [9]. The model combined measurements from routine monitoring sites (PM2.5, NO_2_, and O_3_) and one dedicated measurement campaign (BC) with geographic predictors such as traffic indicators, land use, population density, and satellite‐derived information to predict annual averages for 2010 on a 100 m*100 m grid [9]. Residential addresses during pregnancy were geocoded and linked to the corresponding grid cell of the exposure maps to assign individual‐level exposure estimates.

### 2.3 Data Analysis

Multivariable regression analyses examined the associations between the residential exposure levels obtained for four air pollutants as independent and several clinical parameters as dependent variables. These included: (1) GA, birth weight (linear regression), (2) preeclampsia, EOI, IVH (binomial regression), (3) birth weight in percentiles, ROP grade, and BPD grade (ordinal regression). All analyses were adjusted for potential confounders, including maternal age, preeclampsia, GA, birth weight, EOI, and sex. No significant differences in clinical parameters across recruitment years were observed (Kruskal–Wallis and Chi‐square tests, Benjamin–Hochberg corrected, *q*‐value > 0.05), suggesting no temporal sampling bias. Due to missingness or low incidences, associations with maternal diabetes, maternal BMI, tobacco smoke exposure, and socioeconomic status were not tested. Associations between clinical outcomes and exposure levels reflect on differences between higher‐ and lower‐exposed areas, that is, relative spatial contrasts between locations rather than absolute concentrations, assuming spatial patterns remained temporally stable [9]. Results were presented as per unit change and deemed significant at *p* < 0.05. Samples with missing information were excluded from the respective regression analysis. All statistical analyses were performed in R 4.4.0 using the stats 4.5.0 and MASS 7.3‐60.2 packages.

## 3 Results

Mean levels of ambient concentrations (PM2.5: 15.63 ± 2.02 µg/m³, NO_2_: 24.22 ± 8.58 µg/m³, BC: 1.74 ± 0.46 × 10⁻⁵m⁻¹, O_3_: 85.01 ± 3.84 µg/m³; mean and standard deviation) showed significant correlations (BC and NO_2_ *r* = 0.92, BC and O_3_ *r* = −0.91, PM2.5 and O_3_ *r* = −0.57, NO_2_ and O_3_ *r* = −0.88). When related to air quality reference values, PM2.5 and NO_2_ levels exceeded the EU standards of 2024 [10] and WHO air quality guidelines of 2021 [11] (Table 1).

**Table 1:** OR (95% CI) in maternal exposure to airborne toxicants and description and Pearson correlation coefficients of air pollution concentrations 2010.

		PM2.5	NO_2_	O_3_	BC
**Preeclampsia**	**OR (95% CI)**	**2.58 (1.10–6.02)**	1.09 (0.99–1.20)	0.84 (0.69–1.02)	4.94 (0.85–28.58)
	***p*‐value**	**0.029** *	0.065	0.075	0.075
**Birth weight in percentiles**	**OR (95% CI)**	**1.38 (1.02–1.86)**	0.99 (0.92–1.05)	1.02 (0.88–1.18)	0.90 (0.28–2.93)
	***p*‐value**	**0.038** *	0.666	0.777	0.865
**EU standards and WHO guidelines**	**EU 2024 until 1.1.2030** ^**9**^	10 μg/m^3^ (annual)	20 μg/m^3^ (annual)	120 μg/m^3^ (8‐h)	—
	**WHO 2005** ^**10**^	10 μg/m^3^ (annual)	40 μg/m^3^ (annual)	100 μg/m^3^ (8‐h)	—
	**WHO 2021** ^**10**^	5 μg/m^3^ (annual)	10 μg/m^3^ (annual)	100 μg/m^3^ (8‐h)	—
**Air pollution**	**Min**	9.55 μg/m^3^	11.92 μg/m^3^	68.27 μg/m^3^	1.11 10⁻⁵m⁻¹
	**Median**	16.27 μg/m^3^	22.68 μg/m^3^	85.84 μg/m^3^	1.56 10⁻⁵m⁻¹
	**Max**	19.14 μg/m^3^	57.40 μg/m^3^	90.43 μg/m^3^	3.65 10⁻⁵m⁻¹
**Pearson Correlation**	**PM2.5**	1	**0.65**	**−0.57**	**0.58**
	**NO_2_**		1	**−0.88**	**0.92**
	**O_3_**			1	**−0.91**
	**BC**				1

*Note:* Significant *p*‐values (*p* < 0.05) and statistically significant Pearson correlations are highlighted in bold.

* Indicates associations withstanding the inclusion of multiple pollutants with a VIF ≤ 5.

After adjusting for confounders, elevated residential PM2.5 exposure levels were associated with an increased odds of preeclampsia (OR = 2.58, 95% CI = [1.01, 6.02], *p* = 0.029). In the offspring, elevated residential PM2.5 exposure levels were associated with greater odds of belonging to a higher range of birth weight percentiles (OR = 1.38, 95% CI = [1.02, 1.86]). Both associations remained significant after including multiple pollutants with acceptable colinearity (variance inflation factor [VIF] ≤ 5) into the regression models, indicating that women residing in relatively higher polluted areas were at greater risk of preeclampsia and tended to give birth to neonates with relatively higher weight percentiles. Conversely, the weak inverse association between elevated residential PM2.5 exposure levels and early‐onset infection (OR = 0.67, 95% CI = [0.46, 0.98], *p* = 0.038) was not retained after adjusting for co‐pollutants (variables retained with VIF ≤ 5).

When delineating the effects further by the degree of immaturity, very preterm infants (born ≥ 28 and < 32 weeks GA) were characterized by significantly higher estimated residential PM2.5 (median ratio = 1.05), NO_2_ (median ratio = 1.37), and BC (median ratio = 1.43) exposure levels when compared to infants born with extreme prematurity (< 28 weeks GA). In contrast, residential O_3_ exposure levels were significantly higher in the group of infants born extremely premature (median ratio = 0.97) (Wilcoxon rank‐sum tests, Benjamini‐Hochberg corrected, *q*‐value < 0.05). Between these two groups, only incidences for ROP and BPD severity grades were found to differ significantly (Cochran–Armitage tests; ROP: *Z* = −3.00, *p* = 0.003, *q* = 0.012; BPD: *Z* = −3.51, *p* = 0.0004, *q* = 0.0039).

No other significant associations were found between pollutant exposure levels and detailed parameters characterizing the individual course of complications and exposures after preterm birth (see Methods).

## 4 Discussion

Understanding the complexity of different risk factors driving morbidity development in preterm infants is crucial for preventive and risk monitoring strategies. Exposure to airborne pollutants during pregnancy has been associated with premature birth [4]. In line with the observation of toxicant deposition in the placenta, the pollutants were found to exhibit effects on overall placental growth as well as circulation, endothelial function, and blood coagulation, next to the induction of sustained inflammation and oxidative stress [12, 13, 14]. In line with these studies, we confirmed a higher prevalence of preeclampsia in prematurity cases characterized by elevated estimated residential exposure levels to PM2.5 during pregnancy. When delineating these findings further, future studies should consider both direct effects of prenatal oxidative stress and inflammation on cardiovascular health and hemorrhagic complications [2] as well as via the induction of preeclampsia [15].

Our observation of increased odds for a higher range of birth weight percentiles in preterm infants with increased estimated residential exposure to PM2.5 during pregnancy could indicate metabolic effects, thereby in line with findings in adults where PM2.5 induced impaired insulin‐dependent glucose uptake and diabetes [3, 16].

The seemingly contrasting effects of residential PM2.5 exposure on preeclampsia as well as higher birth weight percentiles associated is in line with studies linking preeclampsia to abnormal fetal growth resulting in both fetal growth restriction [17] as well as increased birth weight [18].

When delineating the effect of immaturity further, we found differing patterns: infants with extreme prematurity were characterized by elevated residential exposure levels to O_3_, while pregnancies ending in premature birth ≥ 28 weeks GA were associated with higher residential exposure levels to PM2.5, NO_2_, and BC. The observation of higher residential O_3_ exposure is broadly consistent with previous studies [19, 20]. The findings indicate the need to stratify for the level of immaturity when investigating the impact of airborne toxicants on neonatal outcome alongside other biological and socio‐environmental determinants.

Despite limitations including cohort size and the need for longer‐term follow‐up data, our study highlights the potential to delineate the effects of environmental pollutants in a well‐characterized, clinically relevant patient population. Future studies are needed to deepen our mechanistic understanding about the impact of airborne toxicants on vascular and metabolic health in early life in a vulnerable patient population.

## Author Contributions

Conception and design of the manuscript was done by Anne Hilgendorff, Caroline Johansson and Benjamin Schubert. The data were acquired by Yvonne Kraus, Caroline Johansson, Tanja Seegmüller, Marie Standl, and Kathrin Wolf. Data analysis and interpretation were performed by Anne Hilgendorff, Caroline Johansson, Benjamin Schubert, Marie Standl, Kathrin Wolf, and Juan David Henao Sanchez. The manuscript was written by Anne Hilgendorff, Caroline Johansson, Benjamin Schubert, Kai Förster, Kathrin Wolf, Marie Standl and Juan David Henao Sanchez. Literature research was done by Anne Hilgendorff, Kai Förster and Caroline Johansson, tables were done by Caroline Johansson. The manuscript was drafted for important intellectual content and reviewed by Anne Hilgendorff, Benjamin Schubert, Kai Förster, Carola Voss, Tobias Stoeger, Kathrin Wolf, Marie Standl, and Andreas W. Flemmer.

## Ethics Statement

LMU Ethic Board #195‐07.

## Conflicts of Interest

The authors declare no conflicts of interest.

## Supporting information

Supporting File

## Data Availability

The data that support the findings of this study are available in the supporting information of this article.
